# Natural history of Endoscopic Third Ventriculostomy followed with high resolution MRI

**DOI:** 10.1186/2045-8118-12-S1-O15

**Published:** 2015-09-18

**Authors:** Miguel Trelles, Ignacio Jusue-Torres, Charles Mitchell, Solomon David, Eric W Sankey, Rigamonti Daniele, Ari Blitz

**Affiliations:** 1 Clínica Delgado, Lima, Peru; 2 Johns Hopkins Hospital, Maryland, USA

## Introduction

Endoscopic third ventriculostomy (ETV) is a common procedure utilized to treat non-communicating hydrocephalus. The purpose of this study is to evaluate the morphologic change seen in ETV in adult hydrocephalus defects over time in with constructive interference in steady state (CISS) high resolution MRI.

## Methods

A retrospective review of all post-surgical ETV high resolution MRIs from July 2009 to March 2015 with sagittal CISS images through the midline was performed. Patients with prior surgeries including ventriculostomy tubes were excluded. The ETV defect size was measured in AP and TV dimension by two experienced users. The intraclass correlation coefficient was calculated.

## Results

34 patients with 98 studies were included. Patients had up to 4 follow up studies up to 4 years after ETV surgery. The average defect size on the immediate post-surgical study was 2.2 x 2.4mm (ellipsoid area of 4.6mm2). Two patients had their ETVs close during the studied time (2/34, 6%). All other patients showed an increase in size of the ETV defect during the studied time. The average increase was of 0.02 mm2/day or 7.3 mm2/year with greater increase in the first post surgical year. Intraclass correlation coefficient between measurement was 0.83 and 0.88 for the AP and TV measurement respectively. Clinical data showed larger defects on last follow-up and higher speed of enlargement in older individuals.

## Conclusion

High resolution MRI with sagittal CISS images is useful in the post-operative evaluation of ETVs. A small percentage closed in the first year of follow up with increase in size of the ETV defect on all other patients suggesting the possibility of a size threshold for continued patency in adults.

